# Effects of salbutamol on exhaled breath condensate biomarkers in acute lung injury: prospective analysis

**DOI:** 10.1186/cc6911

**Published:** 2008-05-30

**Authors:** Oriol Roca, Susana Gómez-Ollés, Maria-Jesús Cruz, Xavier Muñoz, Mark JD Griffiths, Joan R Masclans

**Affiliations:** 1 Intensive Care Department (General Area), Hospital Universitari Vall d'Hebron, Pg. Vall d'Hebron 119-129, C.P. 08035 Barcelona, Spain; 2 Pulmonology Department, Hospital Universitari Vall d'Hebron, Pg. Vall d'Hebron 119-129, C.P. 08035 Barcelona, Spain; 3 Ciber Enfermedades Respiratorias (CIBERES). Carretera de Sóller Km.12-Fundació Caubet-Cimera, C.P. 07110, Bunyola (Mallorca), Spain; 4 Unit of Critical Care, Royal Brompton Campus, Imperial College London, Sydney Street, London, SW3 6NP, UK

## Abstract

**Introduction:**

The benefits of β-adrenergic stimulation have been described in acute lung injury (ALI), but there is still no evidence of its anti-inflammatory effect in these patients. Biomarkers in exhaled breath condensate (EBC) were used to study the effects of salbutamol on lung inflammation in mechanically ventilated patients with ALI.

**Methods:**

EBC was collected before and 30 minutes after administration of inhaled salbutamol (800 μg). The following parameters were measured in the samples: volume obtained, conductivity, pH after helium deaeration, and concentration of nitrites, nitrates and 8-isoprostane. The leukotriene B_4 _concentration was measured after sample lyophilization and reconstitution. Results are expressed as the median (interquartile range).

**Results:**

EBC was obtained from six ALI patients, with a median age of 56 (46 to 76) years. At the time of EBC collection, the Lung Injury Score was 3 (2.3 to 3.1) and the PaO_2_/F_I_O_2 _ratio was 133 (96 to 211) mmHg. A significant increase in deaerated EBC pH was observed after salbutamol administration (7.66 (7.58 to 7.75) versus 7.83 (7.67 to 7.91), *P *= 0.028). Trends toward decreased nitrosative species (18.81 (13.33 to 49.44) μM versus 21.21 (8.07 to 29.83) μM, *P *= 0.173) and decreased 8-isoprostane concentration (11.64 (7.17 to 17.13) pg/ml versus 6.55 (4.03 to 9.99) pg/ml, *P *= 0.068) were detected. No changes in leukotriene B_4 _concentration were found (1.58 (0.47 to 3.57) pg/ml versus 2.06 (1.01 to 3.01) pg/ml, *P *= 0.753).

**Conclusion:**

EBC analysis is a noninvasive technique that can be used to monitor ventilated patients. In EBC from a small cohort of patients with ALI, inhaled salbutamol significantly decreased airspace acidosis, a marker of inflammation, and was associated with a trend toward decreased markers of nitrosative and oxidative stress.

## Introduction

Collection of exhaled breath condensate (EBC) is a novel noninvasive means of obtaining lower respiratory tract samples that can be repeated several times with short intervals between sampling [1]. The collection devices can be used in patients breathing spontaneously as well as in mechanically ventilated patients. The technique is based on the hypothesis that particles exhaled in breath reflect the composition of the alveolar lining fluid. Inflammatory markers and several molecules can be detected in EBC. The concentration of these mediators is influenced by lung diseases and may be modulated by therapeutic interventions; hence, EBC analysis could be a useful, noninvasive technique for monitoring the evolution of lung diseases.

Several studies have reported mediator changes in EBC samples from acute respiratory distress syndrome (ARDS) patients, such as an increased hydrogen peroxide concentration [2] and an increased 8-isoprostane (8-*iso*PGF_2α_) concentration in patients with, or at risk for, ARDS as compared with normal control subjects [3]. A correlation between the EBC nitrite concentration and the tidal volume has been found in acute lung injury (ALI) patients, possibly reflecting ventilator-associated lung injury [4]. The EBC pH has been related to the extent of lung injury, and a good correlation with EBC IL-6 and IL-8 concentrations has been observed [5]. In addition, ALI and ARDS patients show higher EBC cytokine concentrations than healthy volunteers [6].

The potential benefits of β-adrenergic stimulation in ALI include epithelial protection, decreased neutrophil chemotaxis and activation, lower proinflammatory cytokine production, increased surfactant secretion, improved respiratory mechanics, and increased alveolar fluid clearance [7-9]. No studies have been conducted, however, to determine the possible anti-inflammatory effect of β-adrenergic drugs in ALI patients by measuring biomarkers in EBC or plasma, even though studies in healthy volunteers have suggested that salbutamol may be effective for this purpose [10,11].

The aim of the present study was to use EBC biomarkers to investigate whether salbutamol has anti-inflammatory effects on the lungs of mechanically ventilated patients with ALI.

## Materials and methods

### Study population

Mechanically ventilated adult patients who met the criteria for ALI according to the American–European Consensus Conference definition [12] were eligible for participation in the study. All patients were ventilated according to the ARDS Network low tidal volume (ARMA)study protocol [13]. The exclusion criteria were age < 18 years, chronic obstructive pulmonary disease with chronic β-adrenergic treatment, β-adrenergic agents taken within 12 hours before enrollment, unstable asthma, coronary artery disease with a contraindication for β-agonist administration, surgical procedure required within 24 hours before enrollment, immunosuppressive therapy (steroids > 20 mg/day, chemotherapy, or other immunosuppressive agents within 2 weeks), administration of nonsteroidal anti-inflammatory drugs, pregnancy, participation in other interventional trials 30 days prior to enrollment, or allergy to salbutamol.

The study was approved by the local Ethics Committee and informed consent was obtained from the patients' family before inclusion.

### Clinical data

Ventilatory parameters, pulmonary gas exchange, the Lung Injury Score [14], and the Sequential Organ Failure Assessment score [15] were recorded before starting EBC collection. The presence of infection was investigated before starting treatment. Electrocardiographic, hemodynamic, and respiratory parameters were monitored during EBC collection and salbutamol administration.

### Exhaled breath condensate samples

Samples were collected before and 30 minutes after administration of inhaled salbutamol (800 μg: measured pH, 7.0) by metered-dose inhaler (Salbutamol Aldo-Unión EFG, Esplugues de Llobregat, Spain). EBC was collected using a commercially available condenser (EcoScreen; Jaeger, Würzburg, Germany), fitted with an adapter for mechanically ventilated patients (VentAdapter; FILT Lung and Chest Diagnostic GmbH, Berlin, Germany). The heat and moisture exchanger was removed 1 minute before starting EBC collection. The EBC condenser cooled exhaled breath at -20°C. The collector temperature was measured at the beginning of collection. The EBC (1 to 2 ml) was collected in 25 to 45 minutes, depending on the patient's volume per minute. Samples were transferred immediately to the laboratory for processing.

Each EBC sample was divided into 500 μl aliquots in two to four polypropylene tubes (Biosigma, Venice, Italy). The aliquots, used for the measurement of nitrites, nitrates, 8-*iso*PGF_2α _and leukotriene B_4 _(LTB_4_), were immediately stored at -70°C, and were analyzed within 1 month after collection. The other aliquots were used to measure the conductivity and pH before and after deaeration.

### pH and conductivity measurement

The pH was measured in one of the aliquots immediately after deaeration with helium (350 ml/min for 10 minutes), using a model GLP 21 calibrated pH meter (Crison Instruments SA, Barcelona, Spain) with an accuracy of ± 0.01 pH. Conductivity was measured immediately after collection using a model COND 510 conductivity meter (XS Instruments; OptoLab, Milan, Italy) with an accuracy of ± 1%.

### Exhaled breath condensate nitrite/nitrate, 8-isoprostane and leukotriene B_4 _concentrations

The nitrate/nitrite concentration was determined by a colorimetric assay based on the Griess reaction in which sample duplicates were reacted with Griess reagent (Cayman Chemical, Ann Arbor, MI, USA) and were measured at 540 nm absorbance with a microplate reader. The assay sensitivity was 1 μM for nitrite and 2.5 μM for nitrate.

The EBC 8-*iso*PGF_2α _concentration was determined by a competitive enzyme immunoassay using a commercially available kit (Cayman Chemical). The assay sensitivity was 4 pg/ml.

The LTB_4 _concentration in EBC samples was determined by a LTB_4 _EIA kit (Cayman Chemical) after sample lyophilization and reconstitution. The assay sensitivity was 13 pg/ml.

### Statistical analysis

Descriptive statistics are expressed as the median (interquartile range). Differences between groups were analyzed by the Wilcoxon test. Spearman's rank correlation coefficient was applied to determine correlations between the various parameters studied. Significance was set at *P *< 0.05 (two-sided). SPSS 13.0 for Windows (SPSS, Inc., Chicago, IL, USA) was used for the statistical analyses.

## Results

### General characteristics of the study population

Six patients (four males, two females) aged 56 (46 to 76) years were studied; none were current smokers. The origin of ALI was intrapulmonary in five patients. The etiologies included pneumonia (four patients), smoke inhalation (one patient), and extrapulmonary sepsis (one patient). Before EBC collection, patients had been mechanically ventilated for 55 (37 to 76) hours, and evolution of lung injury was 58 (32 to 79) hours.

At the time of EBC collection, the patients' Lung Injury Score was 3 (2.3 to 3.1) and the PaO_2_/F_I_O_2 _ratio was 133 (96 to 211) mmHg. The Sequential Organ Failure Assessment score was 9 (5.8 to 12). No significant changes were observed in the plateau pressure (27 (24 to 30) cmH_2_O versus 26 (21 to 29) cmH_2_O, *P *= 0.416) or in the intrinsic positive end-expiratory pressure (0.5 (0 to 2.5) cmH_2_O versus0.25 (0 to 0.9) cmH_2_O, *P *= 0.18) following salbutamol administration.

Pulmonary gas exchange values before and after salbutamol inhalation are summarized in Table 1. There were no adverse events related to EBC collection or salbutamol inhalation.

**Table 1:** Pulmonary gas exchange values before and after salbutamol administration

	Salbutamol		*P *value
	Before administration	After administration	

PH	7.45 (7.39 to 7.51)	7.43 (7.37 to 7.46)	0.080
PaO_2_/F_I_O_2 _ratio (mmHg)	133 (96 to 211)	140 (102 to 154)	0.600
Partial pressure of arterial carbon dioxide (PaCO_2_) (mmHg)	41 (37 to 48)	43 (39 to 50)	0.072
Bicarbonate (HCO_3_^-^) (mmol/l)	26.5 (23.5 to 32.7)	26 (24 to 32.3)	0.680
Base excess (BE) (mmol/l)	2.4 (-1.4 to 9.1)	2.2 (-0.6 to 8.7)	0.916
Arterial oximetry (SaO_2_) (%)	97 (96 to 98)	97 (96 to 98)	1.000

Results expressed as the median (interquartile range).

### Exhaled breath condensate measurements

The main results of EBC measurement are presented in Table 2. Before salbutamol administration, there was a significant correlation between postdeaeration pH and nitrite levels (*r *= -0.899, *P *= 0.015). A positive correlation was also found between nitrosative species and LTB_4 _(*r *= 0.943, *P *= 0.005), and between nitrosative species and 8-*iso*PGF_2α _(*r *= 0.9, *P *= 0.037). The tidal volume, the PaO_2_/F_I_O_2 _ratio, and the Lung Injury Score showed no correlations with EBC pH or biomarkers.

**Table 2:** Exhaled breath condensate biomarkers before and after salbutamol administration

	Salbutamol		*P *value
	Before administration	After administration	

pH after deaeration	7.66 (7.58 to 7.75)	7.83 (7.67 to 7.91)	0.028
Conductivity (μS)	74 (25.4 to 166)	64 (41.1 to 293.5)	0.465
Leukotriene B_4 _(pg/ml)	1.58 (0.47 to 3.57)	2.06 (1.01 to 3.01)	0.753
Nitrite (μM) + nitrate (μM)	18.81 (13.33 to 49.44)	21.21 (8.07 to 29.83)	0.173
8-isoprostane (pg/ml)	11.64 (7.17 to 17.13)	6.55 (4.03 to 9.99)	0.068

Results expressed as the median (interquartile range).

Deaerated pH values were higher after salbutamol inhalation than before (*P *= 0.028). Total nitrates and LTB_4 _were detectable in EBC samples from all patients, whereas nitrites and 8-*iso*PGF_2α _were detectable in five patients. A tendency to decreased nitrosative species and decreased 8-*iso*PGF_2α _concentrations was observed after salbutamol inhalation (*P *= 0.173 and *P *= 0.068).

## Discussion

In the present small study, we report the apparent anti-inflammatory effects of an inhaled β-adrenergic agent in ALI patients on biomarkers in EBC. A significant increase was observed in the deaerated EBC pH after salbutamol inhalation, as well as a trend to decreased total nitrate and 8-i*so*PGF_2α _concentrations.

The pH of the airway lining fluid is the result of a balance between different buffer systems and the production and release of acids and bases in the airways [1]. In a healthy airway, several factors favor acidification of the airway lining fluid, such as secretion by alveolar type 2 cells and macrophages, necrosis of macrophages and the alveolar carbon dioxide partial pressure (pCO_2_). The pH becomes more alkaline in the proximal airway owing to airway epithelial cell enzyme systems, ion channel activity and buffering proteins [16]. The normal range of EBC pH for healthy subjects is 7.4 to 8.8 [1].

Few studies have reported EBC pH values for mechanically ventilated patients. In otherwise healthy patients undergoing lung resection for cancer, the mean EBC pH obtained using RTubes™ was 7.8 [17]. Another study of patients undergoing cardiothoracic surgery that used the same EBC collection device reported pH values between 5 and 7 [18]. Finally, using the EcoScreen in mechanically ventilated patients, a mean deaerated EBC pH value of 5.98 was reported [5]. In that study, however, the pH measurements were performed at 10°C – in contrast to our study, where the pH was measured when the sample reached room temperature. The condensing equipment and the condensing and measuring temperatures used may all affect the EBC pH [19], making comparison of reported results between studies difficult.

Three essential mechanisms in ALI patients may lead to EBC acidification [5]. Firstly, lactate production is increased in hypoxia due to continuing glucose utilization. Secondly, there is a reduced local buffer capacity secondary to inhibition of glutaminase activity [20]. Finally, neutrophilic inflammation and oxidative stress in the airspace associated with multiple lung diseases [21] has been associated with acidification of EBC that was reversible with anti-inflammatory therapy.

EBC acidity is an important marker of lung inflammation, and the higher EBC pH values detected after a single dose of salbutamol in our study could be related to an anti-inflammatory effect of β-adrenergic stimulation in ALI patients. No significant changes in the partial pressure of arterial carbon dioxide (PaCO_2_) after salbutamol inhalation were observed, which means that changes observed in the pH were not caused by changes in alveolar ventilation. In a recent study, EBC pH values were continuously monitored in mechanically ventilated patients, and EBC acidification was observed even before the clinical alteration appeared [22]. This phenomenon has also been observed in studies on chronic obstructive pulmonary disease [22] and asthma exacerbations [20,21], bronchiestasis [21], cystic fibrosis [23], and other studies on ALI [5,18]. To date there are no reports investigating the possible anti-inflammatory effect of β-adrenergic drugs in ALI patients. The anti-inflammatory effect of this agent has been demonstrated, however, in healthy volunteers undergoing prior lipopolysaccharide inhalation, which generates a neutrophil influx and degranulation into the lungs. This effect was strongly reduced by salmeterol inhalation [10]. Likewise, salbutamol inhibits platelet-activating factor-induced pulmonary neutrophil sequestration [11].

Experimental data have shown that β-adrenergic stimulation can significantly decrease proinflammatory cytokine expression, chemokine mRNA induction, and the resultant neutrophil lung infiltrate in an animal model of endotoxin-induced ALI [24]. More recently, Perkins and colleagues [25] demonstrated that salbutamol can stimulate epithelial repair, and the results of another retrospective study suggest that high doses of salbutamol are associated with shorter duration and less severe acute lung injury [26]. In addition to this potential anti-inflammatory effect, there may be beneficial mechanical effects and improved reabsorption of pulmonary edema. Effectively, it has been shown that airway resistance and peak and plateau pressures decrease, and that dynamic compliance improves with salbutamol administration [27-29]. Moreover, some studies have demonstrated that β-adrenergic stimulation increases alveolar fluid clearance [9,30,31], by stimulating the apical amiloride-sensitive sodium channel and the baseline Na^+^/K^+^-ATPase alveolo-capillary channel, which allows passage of water from the alveoli to the interstitium [32]. Nevertheless, a direct effect of salbutamol inhalation on EBC pH cannot be ruled out, even though there is no published evidence indicating this fact and the measured salbutamol pH was neutral.

A trend towards decreased nitrosative species and 8-*iso*PGF_2α _was observed after a single dose of salbutamol. Few studies to date have used EBC in mechanically ventilated patients, particularly in ALI patients [3-6,18,22]. Several inflammatory mediators are present in EBC samples, such as interleukins [5,6], leukotrienes [18], reactive oxygen [3] and nitrogen species [4]. Pulmonary nitric oxide production has been shown to be stimulated by mechanical forces [33]. Release of pulmonary nitric oxide species may reflect alveolar distension and inflammation. In fact, EBC nitrite has been closely correlated to tidal volume, and the EBC nitrite and tidal volume ratio has been strongly correlated to the extent of lung injury, using the oxygenation criteria of the consensus definition or the Lung Injury Score [4]. The nitrosative species decrease observed in the present study could therefore be related to some improvement in mechanical stress in these patients, even though no significant changes in plateau pressure or intrinsic positive end-expiratory pressure were observed, probably because their values before salbutamol inhalation were normal. 8-*iso*PGF_2α _is a marker of oxidative stress [34] in patients with asthma, interstitial lung disease, chronic obstructive pulmonary disease, cystic fibrosis and ALI [1,3]. Our results suggest that salbutamol inhalation may play a role in preventing the lipid peroxidation that occurs in ALI and ARDS patients, although further study is needed to confirm this hypothesis.

No changes were seen in the EBC LTB_4 _concentration after salbutamol administration, a finding that may have been affected by the small sample size and by the fact that only single-dose administration was tested.

The present study is preliminary and has some limitations related to the EBC technique and to the small size of the patient cohort. The samples obtained are extremely diluted and most biomarkers are at the low end of assay sensitivity. A potential option to overcome this problem is to increase the concentration of the samples, as was done with LTB_4_, with lyophilization being one of the methods of choice [1,5,6]. Furthermore, the relative contribution of the lower respiratory tract versus alveoli in the final composition of the sample obtained is unknown. Nevertheless, in mechanically ventilated patients we minimize one of the main problems of this technique, which is salivary contamination of the sample.

## Conclusion

In summary, EBC is a novel technique that can be used to monitor ventilated patients and to assess therapeutic interventions. In the future, it may have an important role in monitoring ALI patients because it is completely noninvasive and no adverse effects have been described to date. In our small series, inhaled salbutamol significantly increased the deaerated EBC pH and showed a tendency to decrease nitrosative species and the 8-*iso*PGF_2α _concentration in patients with ALI.

## Key messages

• EBC is a novel technique that can be used to monitor airspace inflammation in ventilated patients and to assess therapeutic interventions.

• In ALI patients, higher EBC pH values were detected after a single dose of inhaled salbutamol, which could be related to an anti-inflammatory effect of β-adrenergic stimulation.

• A trend to decreased nitrosative species and 8-*iso*PGF_2α _was observed after a single dose of salbutamol, suggesting that it may play a role in preventing the lipid peroxidation that occurs in ALI and ARDS patients.

## Abbreviations

ALI = acute lung injury; ARDS = acute respiratory distress syndrome; EBC = exhaled breath condensate; IL = interleukin; 8-*iso*PGF_2α _= 8-isoprostane; LTB_4 _= leukotriene B_4_; PaO_2_/F_I_O_2 _= arterial oxygen partial pressure:fraction of inspired oxygen, ratio

## Competing interests

The authors declare that they have no competing interests.

## Authors' contributions

OR conceived the study, carried out the sample collection, performed the statistical analysis and drafted the manuscript. SG-O was involved in the design of the study, carried out the EBC sample measurements and reviewed the manuscript critically for intellectual content. M-JC and XM were involved in the design of the study and reviewed the study critically for important intellectual content. MJDG reviewed the study critically for important intellectual content. JRM conceived the study, carried out the sample collection and gave the final approval to the version to be published.
